# Marmosets: a promising model for probing the neural mechanisms underlying complex visual networks such as the frontal–parietal network

**DOI:** 10.1007/s00429-021-02367-9

**Published:** 2021-09-13

**Authors:** Joanita F. D’Souza, Nicholas S. C. Price, Maureen A. Hagan

**Affiliations:** 1 Department of Physiology and Neuroscience Program, Biomedicine Discovery Institute, Monash University, 26 Innovation Walk, Clayton, VIC 3800 Australia; 2 Australian Research Council, Centre of Excellence for Integrative Brain Function, Monash University Node, Clayton, VIC 3800 Australia

**Keywords:** Marmosets, Neural circuits, Visual attention, Saccades, Optogenetics, Frontal cortex, Parietal cortex

## Abstract

The technology, methodology and models used by visual neuroscientists have provided great insights into the structure and function of individual brain areas. However, complex cognitive functions arise in the brain due to networks comprising multiple interacting cortical areas that are wired together with precise anatomical connections. A prime example of this phenomenon is the frontal–parietal network and two key regions within it: the frontal eye fields (FEF) and lateral intraparietal area (area LIP). Activity in these cortical areas has independently been tied to oculomotor control, motor preparation, visual attention and decision-making. Strong, bidirectional anatomical connections have also been traced between FEF and area LIP, suggesting that the aforementioned visual functions depend on these inter-area interactions. However, advancements in our knowledge about the interactions between area LIP and FEF are limited with the main animal model, the rhesus macaque, because these key regions are buried in the sulci of the brain. In this review, we propose that the common marmoset is the ideal model for investigating how anatomical connections give rise to functionally-complex cognitive visual behaviours, such as those modulated by the frontal–parietal network, because of the homology of their cortical networks with humans and macaques, amenability to transgenic technology, and rich behavioural repertoire. Furthermore, the lissencephalic structure of the marmoset brain enables application of powerful techniques, such as array-based electrophysiology and optogenetics, which are critical to bridge the gaps in our knowledge about structure and function in the brain.

## Introduction

Understanding the neural mechanisms underlying cognitive functions has proven to be a difficult feat for the field of neuroscience. The overwhelming majority of systems neuroscience experiments have focused on the contribution of individual brain areas to a particular behaviour or cognitive process. In practice, no brain area works in isolation. Networks of brain areas work together—wired together with precise anatomical connections—to carry out high-level brain functions. A prime example of this is the frontal-parietal network, which comprises a cluster of areas across the frontal and posterior parietal cortices (PPC), that have been tied to a wide range of behavioural and cognitive processes including oculomotor control, motor preparation, visual attention, and decision-making (Wurtz and Mohler 1976; Colby et al. 1996; Kustov and Robinson 1996). In this review, we focus on two key areas within the frontal–parietal network: the frontal eye fields (FEF) and the lateral intraparietal area (area LIP), both of which have been tied to the control of brief, rapid exploratory eye movements known as saccades. On the surface, both the FEF and area LIP appear to have largely overlapping functional roles, but the contribution of each of these nodes independently, still remains unknown. We have specifically chosen to discuss the LIP-FEF path in association with oculomotor processes, because saccadic eye movements are a simple and effective way of probing visual behaviour and attention. However, the common marmoset (*Callithrix jacchus*), like the rhesus macaque (*Macaca mulatta*) and humans, display coordinated eye–hand behaviours (Hook and Rogers 2008), and areas of their frontal cortex suggest some anatomical similarities to macaque motor and premotor reach and grasp areas (Burman et al. 2014a, b). This suggests that they may also be a suitable model for some visually guided reaching behaviours as well, although with some important caveats (see (Bakola et al. 2015) for a review).

Non-invasive techniques that allow for whole brain studies, such as functional magnetic resonance imaging (fMRI) and magnetoencephalography (MEG) have been useful in looking at network interactions, but are limited either spatially or temporally, making it impossible to draw conclusions at the level of precise anatomical connectivity. While voltage and calcium-sensitive dyes have permitted valuable measurements and analysis of large populations of neural activity at once, these techniques are sub-optimal for investigations into network activity and interactions between cortical areas. In recent years, multi-area electrophysiology studies aimed at addressing this gap by recording neural activity during visual behaviours have become more common (Saalmann et al. 2007; Dean et al. 2012; Siegel et al. 2015; Wong et al. 2016). However, these techniques largely only permit correlational conclusions to be drawn about the neural activity that accompanied cognitive functions. To claim that a particular region plays a causal role in the generation of a cognitive function, the activity of individual neurons must be perturbed to observe the direct effects on cognitive functions and behaviour (Parker and Newsome 1998). Traditionally, this has been achieved through invasive techniques, such as electrical stimulation or cortical lesions, induced via pharmacological manipulations or cooling methods. While these techniques are extremely valuable, they lack the temporal and spatial precision necessary to study functional contributions of specific cell types and their interactions with other brain regions. Often, they non-selectively involve large regions of cortex, simultaneously activating all output pathways in a region. Designer receptors exclusively activated by designer drugs (DREADDs), a technique that can control molecularly defined subsets of cells through engineered G-protein-coupled receptors, overcomes the spatial resolution limitations of these aforementioned techniques, but sacrifices temporal resolution. DREADDs operate in the range of minutes to hours, modulating neuronal activity in a much more prolonged fashion (reviewed in (Whissell et al. 2016). Currently, optogenetics is the only technique with both high temporal and spatial resolution, offering a way in which to target and control precise cell types in vivo, on a millisecond time-scale (Boyden et al. 2005; Deisseroth 2011). By genetically modifying specific types of neurons and causing them to express light-sensitive membrane proteins known as opsins, this technique enables precise, targeted control of neural circuits with specific wavelengths of light that can be delivered from outside an intact dura.

One current limitation in connecting these technologies to visual and cognitive behaviours is the choice of animal model. Rodents have been the primary animal model for the development of new molecular technologies, but they lack homologous cortical networks to humans and rely far less on their vision. Primates, on the other hand, predominantly use their vision and saccadic eye movements to monitor and interact with their environment. The macaque monkey in particular, has been the main source for understanding cognitive visual behaviour, because its cortical networks, including the frontal-parietal, are remarkably similar to humans. However, in macaques, many key areas of interest, including parts of FEF and area LIP, are buried within the sulci of the brain, making access challenging, particularly for precise neural stimulation, multi-electrode laminar recordings, and imaging studies.

**Fig. 1:**
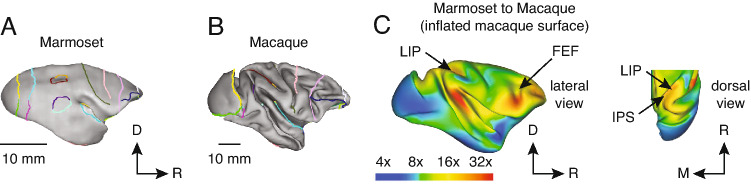
Expansion of frontal–parietal areas are conserved in primate evolution. **A** Marmoset and **B** macaque brains with identified landmarks which served as anchor points to calculate expansion of cortex across species. Notably, the IPS (maroon) and anterior border of area 8aV (lavender) were used. **C** Expansion map projected onto the surface of an inflated macaque brain showing the lateral view of cortex and a dorsal view of the posterior parietal cortex. Color scale indicates the factor of expansion. D, dorsal; M, medial; R, rostral. Adapted from (Chaplin et al. 2013)

Recently, the common marmoset, a small-bodied New World primate, has gained popularity as a primate model for neurophysiology research (Solomon and Rosa 2014; Mitchell and Leopold 2015). Among their many advantages, marmosets have a small body and fast reproduction cycles, making them amenable to transgenic technology (Sasaki et al. 2009). The anatomical subdivisions of cortical areas in the marmoset have also been mapped, and their anatomical connections are consistent with humans and macaques (Rosa et al. 2009; Majka et al. 2016, 2020). Furthermore, marmosets have a rich behavioural repertoire consisting of both natural (Miller and Wren Thomas 2012) and conditioned (Mitchell et al. 2014, 2015) tasks. Marmosets also rely strongly on their sense of sight—a fact that is reflected in the large fraction of their neocortex dedicated to visual processing (Rosa et al. 2009; Majka et al. 2016, 2020) and their highly developed visual system that is homologous to higher-order primates like humans and macaques (Chaplin et al. 2013; Zhu and Vanduffel 2019). Compared to humans and other primate models, marmosets have a relatively small brain size, which may be a limitation in their use for studying cognitive behaviour. However, in the evolutionary development of primate brains, the differential expansion of cortical areas has largely been conserved across primate species relative to brain size (Chaplin et al. 2013; Zhu and Vanduffel 2019). This means that changes in cortical expansion from marmoset to macaque in a given cortical area (Fig 1A, B) are predictive of the expansion expected from macaque to human. Some areas of cortex have expanded greatly with brain size, such as the ventrolateral prefrontal cortex and temporal parietal junction, which in humans are related to high-level cognitive functions including speech and communication (Fig 1C, red regions). Conversely, areas of the PPC (containing area LIP), scale more modestly between marmosets and macaques (Fig 1C, dorsal view, arrow), and the expansion of early-developing visual areas like V1 is even more modest (Fig 1C, dorsal view, dark blue regions). This suggests that marmosets may still be a useful model for studying many visual behaviours, despite their small brain size.

Recent research has also confirmed that marmosets can be trained to perform a wide range of visual, oculomotor and cognitive tasks while head restrained, in a manner comparable to macaques and humans (Mitchell et al. 2014, 2015; Johnston et al. 2019; Cloherty et al. 2020). The marmoset brain also has major advantages over rodents and other primate models for neurophysiological research. Critically, the surface of the marmoset brain is lissencephalic, exposing nearly all of its visual cortex on the lateral surface just below the skull, enabling direct access to many high-level brain areas—including important visual and oculomotor areas—that do not have clear homologues in the rodent and that are otherwise buried deep in sulci in primates like the macaque. This facilitates optical imaging and permits precise perpendicular penetrations of cortical layers for laminar analysis of local microcircuits in higher-order visual areas, such as FEF and area LIP, that are normally hidden in sulci. It also enables large-scale neuronal recordings over entire cortical areas, uninterrupted by sulci and permits access to multiple cortical areas simultaneously (Isa 2017). As such, marmosets serve as an important experimental bridge by which advances in the mouse community can be applied to the primate brain.

Acknowledging that the viability of marmosets for studies on the visual system has been previously reviewed (Solomon and Rosa 2014; Mitchell and Leopold 2015), here, we extend upon and update this body of knowledge, by specifically examining why marmosets are an ideal model for untangling the neural mechanisms underlying inter-area interactions in the visual system. Using the frontal–parietal network as an example in marmosets, we will explore what is currently known about the structure and function of key nodes in this network, and how emerging technology, such as optogenetics, may be used to dissect the function of neural circuits.

## Key areas in the frontal–parietal network are conserved in marmosets and macaques

Areas of the frontal–parietal network have been largely defined based on function, and do not precisely overlap with cyto-architecturally defined boundaries. Furthermore, sulcal folding in the brains of higher-order primates, such as humans and macaques, makes precise anatomical reconstructions challenging. For example, FEF in primates is defined functionally by the ability to induce and influence saccades via micro-stimulation. In macaques, FEF is likely composed of cytoarchitectural areas 45, 8aV, and 6 (Bruce et al. 1985; Stanton et al. 1989; Schall et al. 2020), which have anatomical connections with the parietal and visual cortices (Schall et al. 1995) and connections with the superior colliculus both directly (Fries 1984) and via the pulvinar (Sommer and Wurtz 2006; Berman et al. 2009). Projections from FEF to the PPC are dense, feedback projections (Stanton et al. 1995; Schall et al. 1995). Area 8aV is characterized by large pyramidal neurons in layer V, and at least partially overlaps with the region in which micro-stimulation evokes saccadic eye movements (Stanton et al. 1989). In marmosets, the precise anatomical boundaries of FEF are still being established. However, converging evidence suggests that a similar group of cytoarchitectural areas is involved. Anatomically, areas 8aV and 6DR form monosynaptic reciprocal connections with extra-striate visual areas and the posterior parietal cortex (Burman et al. 2006; Reser et al. 2013). One anatomical tracing study has identified neurons in the frontal cortex of the marmoset that project to the superior colliculus (Collins et al. 2005). The region appears to correspond roughly to marmoset area 8, however, this study did not register to precise cytoarchitectural areas and their injections spanned superficial to deep layers of the superior colliculus. More detailed anatomical studies are needed to fully map out the marmoset cortico-collicular circuits associated with oculomotor behaviours.

In 1874, Ferrier and colleagues were the first to unilaterally stimulate FEF in anaesthetised macaques and elicit rapid contralateral eye movements known as saccades (Ferrier 1874). This general finding that a brief stimulation of FEF produces a single contralateral saccade, of a particular amplitude, direction, latency and threshold, was reliably replicated by several other groups in the coming years, and in both humans and awake non-human primates (Robinson and Fuchs 1969; Wurtz and Mohler 1976; Bruce et al. 1985; Schmolesky et al. 1998; Schall et al. 2020). One notable study stimulated FEF in over 300 locations and noticed that the amplitude and direction of saccades changed in a stereotyped pattern, depending on where and how FEF was stimulated (Robinson and Fuchs 1969). Evoked saccades in FEF also follow a visual topography — smaller, more foveal saccades are evoked from stimulation of the ventrolateral regions and larger, more peripheral saccades in the dorsomedial regions of FEF (Bruce et al. 1985).

**Fig. 2:**
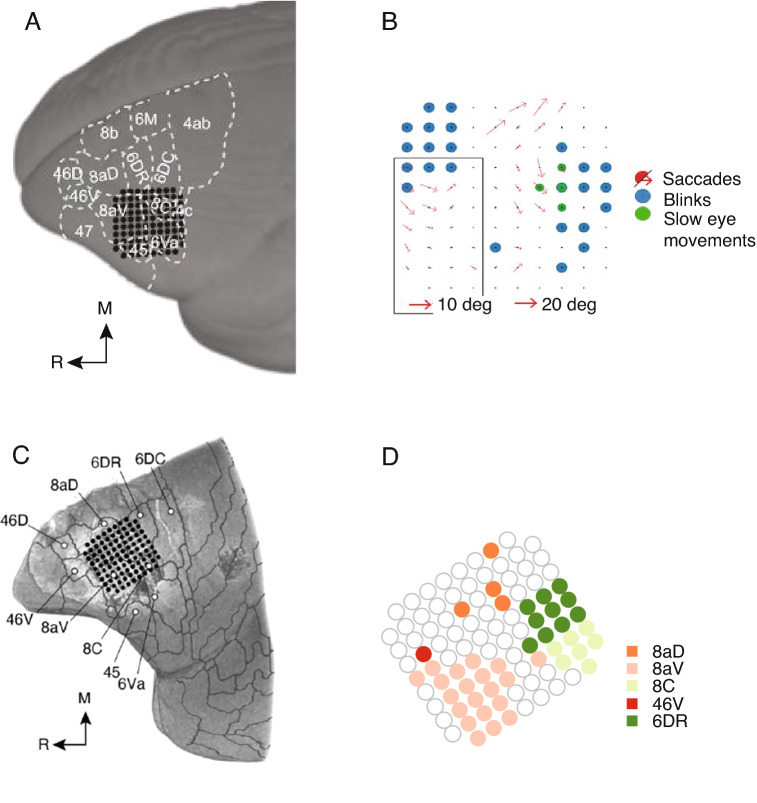
FEF-like responses in the marmoset prefrontal cortex. **A** Example marmoset MRI reconstruction of array recording locations aligned to cytoarchitectural boundaries and **B** Electrode map for example showing location of saccade, blink and smooth pursuit responses in response to microstimulation. Red arrows indicate amplitude of evoked saccades. Adapted from (Selvanayagam et al. 2019). **C** Example marmoset MRI reconstruction of array recording locations aligned to cytoarchitectural boundaries and **D**, Electrode map showing location of visual responses according to cytoarchitectural area. Adapted from (Feizpour et al. 2021). M, medial; R, rostral

Consistent with macaque literature, electrical stimulation of several frontal cortical sites in marmosets evokes eye and head movements (Mott et al. 1910; Blum et al. 1982). Recently, the dorsolateral prefrontal cortex of awake, free-viewing marmosets was electrically stimulated using a 96-channel Utah array (Selvanayagam et al. 2019). By co-registering their recordings with anatomical MRIs, they were able to provide precise reports of cytoarchitectural areas. In addition to evidence placing marmoset FEF in a similar relative location within oculomotor areas 45 and 8aV (Burman et al. 2006; Reser et al. 2013; Schaeffer et al. 2019), Selvanayagam and colleagues (2019) found that like human (Silver and Kastner 2009; Jerde and Curtis 2013) and macaque FEF (Bruce et al. 1985; Schall et al. 1995), marmoset FEF is organised in a topographical manner, according to saccade direction and amplitude. Area 45 and the lateral portion of 8aV were associated with smaller, more foveal saccades (Fig. 2A, B), and saccade amplitudes increased medially across the array, spanning areas 8aV and 6DR. In marmosets, the saccades evoked by area 6 stimulation tend to be goal-directed in contrast to vector coding of area 8aV, which is similar to the cortical eye fields in macaques (Selvanayagam et al. 2019). Among the few differences between marmosets, humans and macaques, saccade latencies in marmosets at low currents were noted to be longer and more variable. It is worth noting that there is some discrepancy in the cytoarchitectural definition of area 45 between humans and monkeys. Both macaque (Schall et al. 1995) and marmoset (Selvanayagam et al. 2019) studies have suggested that as small amplitude saccades can be evoked from area 45, it likely comprises the ventral portion of the FEF. Area 45 in these studies is defined using Walker’s definition (Walker 1940), as large pyramidal cells in layers III and V (Schall et al. 1995; Burman et al. 2006; Reser et al. 2013). In humans, area 45 is characterized by large pyramidal cells in layer III but not layer V (Petrides and Pandya 2002). Cells in area 45 by this definition likely do not have non-oculomotor functions (Petrides et al. 2005).

In addition to evoking saccades, a subset of macaque FEF neurons have visual responses, with large receptive fields (Wurtz and Mohler 1976; Cavanaugh et al. 2012) and brisk response latencies (Mohler et al. 1973; Thompson et al. 1996; Schmolesky et al. 1998). Despite FEF’s relatively late position in the visual hierarchy, visual responses in FEF have short latencies, comparable to early visual areas such as visual area 2 (V2), which may be due to direct connections with the visual cortex or connections that bypass the hierarchy from the superior colliculus via the thalamus. Similar visual responses can be found in marmosets, particularly in areas 8aV, 8C and 6DR (Fig. 2C, D; (Feizpour et al. 2021)). In macaques, the lateral portion of FEF receives afferents from more foveal representations of visual cortex, while the medial portion receives afferents from peripheral representations of visual cortex (Schall et al. 1995). The same is true for area 8aV in marmosets (Reser et al. 2013). Consistent with this, in marmosets, the central visual field was better represented by the lateral aspect of area 8aV and the peripheral visual field was better represented by the medial aspect of area 8aV (Feizpour et al. 2021).

The FEF is not the only specialised region of the frontal–parietal network that contributes towards visual behaviours. In the PPC, area LIP has been implicated in guiding saccadic eye movement control (Andersen et al. 1985, 1990b). Specifically, area LIP neurons respond to the presentation of behaviorally-relevant visual stimuli in localised regions of space (Gottlieb et al. 1998; Kusunoki et al. 2000) and saccade planning (Mazzoni et al. 1996). In macaques, area LIP is buried in the lateral bank of the intraparietal sulcus within the PPC, and has direct reciprocal anatomical connections to other nodes in the saccade control network such as FEF and the superior colliculus (Huerta et al. 1987; Andersen et al. 1990a; Blatt et al. 1990; Schall et al. 1995; Stanton et al. 1995; Paré and Wurtz 1997; Anderson et al. 2011). In fact, micro-stimulation of FEF in macaques elicits an enhanced fMRI activation of area LIP neurons (Premereur et al. 2013) during visually guided saccade tasks, fixation tasks and even in absence of any visual stimulation (Ekstrom et al. 2008). The laminar distribution of neurons and the dorsal–ventral subdivisions and myelination patterns of marmoset area LIP are similar to those in macaques (Rosa et al. 2009). Anatomical studies in marmosets have also triangulated connectivity between area LIP, FEF and the superior colliculus, reflecting observations in the macaque visual system (Collins et al. 2005; Reser et al. 2013; Ghahremani et al. 2017).

Like FEF, micro-stimulation of area LIP neurons evokes eye blinks and saccadic eye movements of a particular direction and amplitude in macaques (Shibutani et al. 1984; Thier and Andersen 1998; Hanks et al. 2006) and marmosets (Ghahremani et al. 2017). Electrophysiological recordings in macaque area LIP have also revealed that most neurons discharge in the ‘planning stage’, just prior to the execution of saccades towards visible and remembered visual targets (Gnadt and Andersen 1988; Barash et al. 1991; Colby et al. 1996; Meister et al. 2013) within their response fields. For example, during saccade tasks where the end target onset is delayed after the fixation target disappears, a “gap effect” of shorter saccade reaction times coupled with an increase in neural activity during the gap period has been recorded in macaques (Chen et al. 2013, 2016) as well as marmosets (Ma et al. 2020). Generally, area LIP neurons respond to a combination of visual stimuli, eye position and the direction and amplitude of planned eye movements, to encode the location of salient visual stimuli in eye-centered or head-centred coordinates (Andersen et al. 1985, 1990b).

Neural activity in the PPC, and notably area LIP has also been shown to be modulated by cognitive factors like visual attention (Goldberg et al. 1990; Colby et al. 1996; Snyder et al. 1997; Corbetta et al. 1998; Colby and Goldberg 1999; Bisley and Goldberg 2003, 2010), reward (Platt and Glimcher 1999; Sugrue et al. 2004) and decision-making (Gold and Shadlen 2000; Wong et al. 2016; Hawellek et al. 2016). Area LIP is thought to integrate bottom–up sensory and top–down cognitive factors by combining and transforming visual information such as initial eye position with cognitive factors to produce a salience representation or ‘priority map’ of the visual field, that combines salient visual features with behavioral goals (Gottlieb et al. 1998; Bisley and Goldberg 2010; Fiebelkorn and Kastner 2020; Chen et al. 2020). In macaques, neurons in area LIP signal the location of the visual target rather than the saccade goal (Gottlieb and Goldberg 1999), which has been suggested to dissociate between the locus of visual attention and the saccade motor plan. However, when visual presentation of the target and saccade execution were temporally separated, it was found that area LIP neurons first encoded the location of the visual cue, and later, some neurons also responded during saccade execution (Zhang and Barash 2000, 2004).

Whether marmoset PPC is also modulated by such cognitive factors is yet to be determined. However, there is evidence that marmosets can perform anti-saccades, a type of cognitively demanding visual task. Anti-saccade tasks typically require subjects to suppress a response to a salient, peripherally appearing stimulus, in favour of saccading to a featureless, unmarked location opposite this salient stimulus (Munoz and Everling 2004; Antoniades et al. 2013). In such tasks, the shape and/or colour of the central fixation point instructs the subject about whether the trial requires a saccade towards (pro-saccade) or away from (anti-saccade) the salient peripheral stimulus. Recently, (Johnston et al. 2019) observed that marmosets were capable of completing a slightly modified version of the traditional anti-saccade task, and that neurons in area LIP responded to both pro- and anti-saccade targets.

## Marmosets are capable of a wide range of visual behaviors

Visual attention and saccadic eye movements are intimately linked from behavioural and anatomical perspectives. The spatial allocation of attention is tightly time-locked and precedes saccade execution (Filali-Sadouk et al. 2010), and to make a saccade towards an appropriate location, attention must be directed towards the spatial location of the saccade target. Anatomically, visual attention and saccadic behaviours recruit a common network in the primate visual system (reviewed in (Wardak et al. 2011)). In humans, fMRI has demonstrated activation of similar regions in the frontal and parietal cortices during saccades and visual attention shifts (Corbetta 1998; Nobre et al. 2000; Perry and Zeki 2000; DeSouza et al. 2003; Munoz and Everling 2004; de Haan et al. 2008).

Given the functional and neuro-anatomical similarities between marmosets and higher-order primates like macaques and humans, it is worth examining whether marmosets are an appropriate behavioural model for probing the frontal–parietal saccade network. For decades, macaques were the ideal model for psychophysical and behavioural paradigms in visual research, demonstrating an immense capacity to learn complex rules and concentrate for long periods of time. Marmosets, on the other hand, have fallen short of macaques on such behaviours—managing to complete approximately half as many trials as macaques, with sessions typically lasting 1–2 h (Mitchell et al. 2014; Hung et al. 2015; Chen et al. 2021). While this can largely be attributed to the type of task being relatively unnatural for marmosets, requiring extended fixation periods and head restraint, we are still in the early stages of learning the extent of their behavioural repertoire. However, with constant improvements in the efficiency and precision of neural data collection and our ability to combine data across sessions, this is no longer a significant limitation to the marmoset model. Marmosets also tend to excel under unrestrained conditions, and in natural free-viewing or visual discrimination tasks that are more actively engaging (Mitchell et al., 2014). As such, marmosets are the ideal candidate when utilizing such paradigms, especially considering the potential for using standard eye tracking to measure visual behaviour in freely moving subjects (Jendritza et al. 2021).

When considering performance under head restraint, the main sequence of saccadic eye movements in marmosets is similar to that observed in macaques and humans, with a linear relationship between the amplitude and peak velocity of saccades (Mitchell et al. 2014; Chen et al. 2021). However, the range to which marmosets can rotate their eyes away from a default central position, is far more restricted compared to macaques and humans (Mitchell et al. 2014; Chen et al. 2021). Marmosets make saccadic eye movements within ten degrees of their initial rest position (Mitchell et al. 2014). Express saccades, or saccades with very low latencies, can be elicited using a ‘gap’ saccade task, in which the central fixation point disappears prior to the saccade target onset. Humans, macaques, and marmosets all exhibit low-latency express saccades in a gap-saccade task (Ma et al. 2020; Chen et al. 2021). Marmosets can also perform smooth pursuit eye movements, in which the eye voluntarily tracks a moving target, with similar velocity and acceleration profiles to macaques and humans (Mitchell et al. 2015). In a motion-discrimination task, marmosets displayed similar speed-accuracy trade-offs to humans. Saccade errors and reaction times increased as the motion signal decreased (Cloherty et al. 2020). Together, these results show that the marmoset is a promising model for studying eye movement behavior.

As previously mentioned, marmosets are capable of completing a slightly modified version of the traditional anti-saccade task (Johnston et al. 2019). In this version of the task, anti-saccade trials were presented in an entirely separate block from pro-saccade trials, and marmosets made anti-saccades towards a small, dimly lit peripheral stimulus. This version of the anti-saccade task has not only been used in human studies (Barton et al. 2002; Edelman et al. 2006; Dafoe et al. 2007; Antoniades et al. 2013), it is also usually the final training stage for macaques—though (Johnston and Everling 2011) have previously noted that some macaques have also struggled advancing past this stage. While this version of the anti-saccade task did not require a geometric calculation of vector inversion of the location of the stimulus into a saccade command, it retained the most important clinical and cognitive components, such as response suppression, voluntary saccade generation, longer reaction times and higher error rates. These core components have been shown to require inhibition of the frontal–parietal saccade network (O’Driscoll et al. 1995; DeSouza et al. 2003).

Saccadic eye movement behaviors are closely linked to cognitive behaviors such as decision-making and visual attention. The extent to which marmoset saccade behavior is linked to cognitive behavior is still poorly understood. However, their eye movements are drawn to salient features of a visual stimulus, similar to macaques and humans. Primates tend to fixate salient features of an image, which may be driven by properties of the visual stimulus like color, luminance and motion (Chen et al. 2021) or be cognitively salient, like faces (Mitchell et al. 2014). Marmosets have also been proposed as a model for studying social behaviors (Miller et al. 2016; Nummela et al. 2017). In a free choice task, where marmosets choose between two saccade targets, marmosets were more likely to choose a target if they first viewed an image of a conspecific, with gaze oriented to one of the target locations (Spadacenta et al. 2019). This ‘joint-attention’ or reflexive gaze following behavior is also observed in humans and macaques, further illustrating that cognitive visual behaviors are homologous in the marmoset.

However, it is important to note that, to date, there has been little evidence that marmosets are capable of the same level of cognitive control as their macaque counterparts. Macaque studies have relied on training subjects to fixate for long durations (on the order of 2–3 s) while suppressing saccades to salient stimuli, whether this is a briefly flashed stimulus in a memory-guided saccade task (Andersen et al. 1990b), a stimulus cueing an anti-saccade away from the stimulus (Gottlieb and Goldberg 1999), or task that requires covertly searching an array of stimuli (Thompson et al. 1996; Wardak et al. 2006). To date, marmoset studies have yet to demonstrate delayed responses to visual stimuli. Fixation durations have been shorter (approximately 1 second, for example in (Ma et al. 2020)). The shorter fixation duration makes delayed-saccade tasks more challenging. Furthermore, it is still an open question whether marmosets can suppress saccades to peripheral targets for more than a few hundred milliseconds (Mitchell et al. 2014). As described above (Johnston et al. 2019), researchers settled on a modified version of the anti-saccade task. While the limits of marmoset behavioural training are still largely unknown, it is important to note that there are likely significant limitations on the types of cognitive visual tasks they can perform compared to macaques.

## Bridging the gap: from brain networks to behavior

**Fig. 3:**
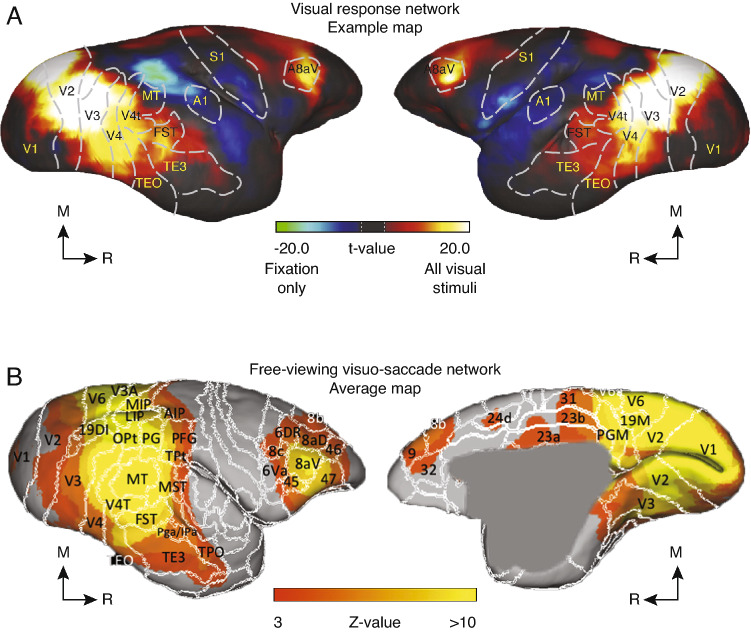
Networks for visual and saccade responses in the marmoset. **A** Visual response network in an example marmoset (left and right hemispheres shown). Functional MRI responses were compared between viewing static visual images and a fixation task (lateral, left and medial, right views shown). Adapted from (Hung et al. 2015). **B** Visuo-saccade network averaged across responses of three marmosets (l. Functional MRI responses were recorded while marmosets free-viewed videos displayed at different locations on a monitor. Adapted from (Schaeffer et al. 2019). M, Medial; R, Rostral

The next frontier in systems neuroscience is understanding not just how individual areas of the brain contribute to cognitive behavior, but how areas of the brain work together. Non-invasive imaging techniques, such as fMRI have the advantage of measuring physiological changes across the whole brain simultaneously. Functional imaging studies have revealed that FEF and area LIP are almost always co-activated in attention and saccade tasks in both humans (Corbetta 1998; Nobre et al. 2000; Perry and Zeki 2000; de Haan et al. 2008) and macaques (Koyama et al. 2004; Baker et al. 2006; Wardak et al. 2010). In marmosets, fMRI has revealed homologous networks for vision (Fig 3A) and saccadic eye movements (Fig 3B). A free-viewing task, in which videos of action movie trailers were displayed at different locations on a screen, revealed activation of visual cortical areas as well as frontal–parietal areas, with peaks in area LIP and area 8aV. The same task in humans confirmed that activation peaked in area LIP and the FEF (Schaeffer et al. 2019). These findings built on an earlier study which found similar results while marmosets viewed static images, although the static images did not evoke as much parietal activity (Hung et al. 2015). Together, these studies lay the foundation for establishing homologous functional networks for visual behavior in the marmoset. Unfortunately, due to the long time-scale of the hemodynamic response, fMRI alone cannot provide insights into the cellular-level circuitry involved in inter-area communication.

In macaques, a growing number of studies have used multi-area extracellular recordings to look at temporal correlations in activity across nodes of the frontal–parietal network. These studies have given insights into how the timing of neural activity across nodes of the network contribute to behavior. For example, neurons in the frontal and parietal cortex signal task and choice information with the same latency (Siegel et al. 2015). Furthermore, temporally coherent neural activity across nodes of the frontal–parietal network is instrumental in guiding eye–hand coordination (Dean et al. 2012), decision-making (Pesaran et al. 2008; Wong et al. 2016) and visual attention (Buschman and Miller 2007; Saalmann et al. 2007, 2012; Gregoriou et al. 2009; Bastos et al. 2015; Fiebelkorn et al. 2018). However, large-scale optical imaging, multi-electrode and multi-area recordings are challenging in macaques, because they rely on access to the entire surface of the cortical areas in question, and most high-level extra-striate areas of the macaque visual system, like FEF and area LIP, lie partially obscured within sulci. Most imaging and multi-electrode studies in macaques have been conducted on earlier visual areas like V1, because they lie exposed and unobscured on the cortical surface (for example see (Chen and Seidemann 2012)) . Typically, such studies have found a homogeneous topographical layout of the visual field across these early visual areas, where neurons in adjacent columns have receptive fields that overlap regions of the retina. However, growing evidence suggests a more fractured and twisted topography exists in extra-striate areas. Notably, the lissencephalic marmoset cortex, which is uniquely suited to such large-scale optical imaging and multi-electrode investigations, has corroborated such evidence, finding a more twisted topography with regions of rapid change in receptive field positions in extra-striate regions like the dorsomedial area (Yu et al. 2020). High-resolution fMRI maps of the macaque visual cortex have also depicted similar heterogeneous topographical layouts and retinotopic maps (Zhu and Vanduffel 2019). The middle temporal area, an area of the brain involved in encoding visual motion, is another example of an area obscured in macaque sulci, but exposed to the cortical surface in marmosets. High-density electrode array recordings from the middle temporal area of the marmoset have revealed population dynamics including neural correlations (Solomon et al. 2015), spatial encoding (Chen et al. 2015), and motion adaptation (Zavitz et al. 2016). Although they did not include population analyses, similar multi-electrode arrays have been used in both the frontal (Selvanayagam et al. 2019; Feizpour et al. 2021) and parietal (Ma et al. 2020) cortices of marmosets.

Furthermore, the marmoset lissencephalic cortex is well suited for linear electrode arrays which enable simultaneous recordings across cortical layers, when inserted perpendicular to the cortical surface. Such recordings are of importance when studying inter-area networks, as a canonical microcircuit has been traced within several neocortical areas, dictating how information is hierarchically processed in a sequential manner (Wiesel et al. 1974; Douglas et al. 1989; Douglas and Martin 2004; Shepherd 2011). Feedforward inputs from regions lower in the visual hierarchy synapse onto the intermediate layer and project up via excitatory neurons to the superficial layers, where they are then integrated with and modulated by feedback information from the same or higher-order cortical regions, before finally projecting to deeper layers (Malach et al. 1997; Solomon et al. 2002; Majaj et al. 2007) and ultimately projecting out to other cortical areas (Thomson and Bannister 2003). In macaque areas that lie on the cortical surface, such as area visual area 4 (V4), laminar-specific mechanisms have been revealed for visual attention (Nandy et al. 2017) and attention modulated V1–V4 communication in directed, laminar-specific manner (Ferro et al. 2021). Unfortunately, many areas of interest in the frontal–parietal network of macaques, including area LIP and parts of FEF are buried in sulci. It is possible to approach the macaque sulcal cortex from an angle that traverses all layers within a column (Schroeder et al. 1998), and a handful of studies have demonstrated the importance of this technique. For example, visual information varies across cortical layers in FEF (Chen et al. 2018) and the level of a subject’s consciousness modulates laminar-specific activity between the thalamus, the FEF and area LIP (Redinbaugh et al. 2020). The marmoset offers a huge advantage in this research space, as a larger proportion of the visual cortex is exposed to the surface, including frontal and parietal areas. There are early examples of marmoset studies assessing cortical areas not accessible in the macaque for laminar recordings. For example, in the marmoset middle temporal area, such arrays have revealed different mechanisms of motion encoding in superficial and deep layers (Solomon et al. 2017). In marmoset frontal area 8aD, laminar electrodes have been used to identify potential inhibitory mechanisms in an anti-saccade task (Johnston et al. 2019). As electrode technologies are rapidly evolving, marmosets are well positioned to address questions related to large-scale, multi-area population dynamics.

Extracellular electrophysiology alone has its limitations. Given that FEF and area LIP both receive input from several extra-striate visual areas (Andersen et al. 1990a; Schall 1991), conclusions about the temporal coherence of neural activity between areas of the frontal–parietal network are limited. It is difficult to dissociate functionally relevant signals from irrelevant ones, and to determine whether coherent fluctuations in two regions are simply due to common input. Furthermore, it is difficult to ascertain directionality in the flow of information across areas. Consequently, there is a need to perturb activity in one area, while recording from the other, to better understand the interactions between directly connected areas. One way to address this is by observing functional changes in a network associated with inactivation (pharmacologically or through cooling) or electrical micro-stimulation. Inactivating either FEF or area LIP (via cooling) while recording from the other changed the firing rate of over 70% of neurons during a memory-guided saccade task (Chafee and Goldman-Rakic 2000). Neurons in each area were equally likely to have firing rates augmented or suppressed, suggesting a fairly equal exchange of information across areas, while neural latencies to the cue response remained unchanged. Likewise, micro-stimulation of FEF results in widespread activation of neurons in area LIP (Ekstrom et al. 2008). Inactivation of both area LIP (Wardak et al. 2002) and FEF (Wardak et al. 2006) results in an increase in reaction time for the selection of a saccade target amongst distractors. However, the deficits increased with task difficulty only in area LIP (Wardak et al. 2002), suggesting a role in encoding visual salience. Representations of visual salience emerge earlier in area LIP than in FEF (Buschman and Miller 2007), suggesting that these signals may propagate from parietal to frontal cortices. Indeed, a recent study found that representations of visual salience in FEF disappeared when the PPC was pharmacologically inactivated (Chen et al. 2020). While these studies have provided invaluable insights, both inactivation and micro-stimulation are spatially crude and non-selectively engage large regions of cortex around the site of stimulation or inhibition. As a result, any observed effects from these techniques could encompass indirect pathways through neighbouring cortical regions. Furthermore, inactivation studies are temporally limited by the time course of the drug or cooling procedure. Therefore, there is a need for techniques that can manipulate neural activity with both spatial and temporal precision to tie neural activity of individual cells to the anatomical projections across areas.

## Optogenetics as tool for dissecting neural circuits

Optogenetics offers a way to modulate direct functional links between anatomically connected areas through precise stimulation or inhibition of specific cell types within an area, while recording from another (Boyden et al. 2005). The technique operates under the same general principle of electrical stimulation, chemical cooling or chemical lesioning agents, but targets specific types of neurons made to express light-sensitive ion channels or pumps, called opsins, to change the polarisation of the cell in response to specific wavelengths of light (Deisseroth 2015). Commonly, illumination of bacteriorhodopsins and halorhodopsins results in hyperpolarisation, which inhibits neural activity, while illumination of channelrhodopsins typically results in depolarisation and action potentials. Briefly, these microbial opsin genes, incorporated into stable and non-replicating viral vectors such as adeno-associated viral (AAV) vectors, are injected into regions of interest, where they are able to efficiently transduce specific types of post-mitotic neurons in the vicinity of the injection. Optogenetic techniques have been predominantly implemented in mouse models, however, their use in primates is growing (as reviewed in (El-Shamayleh and Horwitz 2019) and (Diester et al. 2011)). By exploiting viruses incorporating cell-specific promoters, it has been possible to determine the contribution of different cell types, particularly excitatory and inhibitory cells (Cardin et al. 2009; De et al. 2020). This is important because computational modelling has suggested that temporally coherent neural activity is driven by windows of neural excitation and inhibition (Shewcraft et al. 2020). This opens the door to using optogenetics to probe network dynamics and their cellular origins. Rodent models have already been used to investigate how projections from the frontal cortex to thalamus modulate visual attention (Wimmer et al. 2015; Schmitt et al. 2017). However, the neurophysiology and behaviour of rodents and primates differ in many ways, making it difficult to translate discoveries in rodents to higher-order primates.

In macaques, optogenetics has been used to reveal causal links between neural activity and cognitive behaviours in a manner that effectively extends the result of previous electrical micro-stimulation studies. For example, in macaques trained to saccade to a salient target among distractors, optogenetic stimulation of area LIP increases the number of saccades to targets within the stimulated neurons’ receptive field, and decreases those saccades’ latency (Dai et al. 2014). (Gerits et al. 2012) observed a similar decrease in saccade latencies when optogenetically stimulating the arcuate nucleus in macaques performing a visually guided saccade discrimination task, but reported little to no change in performance accuracy or other saccade metrics such as amplitude. Optical stimulation of V1 neurons transfected with channelrhodopsin (ChR2) variants can generate saccades towards the receptive field of stimulated neurons in macaques trained to perform saccade-dependent visual discrimination tasks (Jazayeri et al. 2012). However, optogenetic stimulation of saccade modulation centres like FEF in macaques (Ohayon et al. 2013; Inoue et al. 2015), is often suboptimal at evoking eye movements with metrics identical to visual or electrical stimulation protocols. Optogenetically evoked saccades in macaques often do not reach the desired eccentricity and are not entirely accurate in their direction trajectories. This is because it is difficult to target all relevant cells in these areas, as optogenetic injections activate a smaller volume of tissue than electrical micro-stimulation and because much of the area is buried in the arcuate sulcus in macaques, making targeting optical stimulation difficult.

In macaques, optogenetic stimulation of FEF axon terminals in the superior colliculus can generate saccades towards the receptive field of stimulated FEF cells (Inoue et al. 2015), indicating that technique can be used to determine the influence of long-range projections. However, the latencies of these optogenetically evoked saccades (Inoue et al. 2015) appear to be 150–170 ms longer than saccades evoked by electrical micro-stimulation (Schiller and Stryker 1972; Bruce et al. 1985). This may be explained by the fact that optical suppression of axon terminals can have unintended consequences if suppressive opsins affect synaptic transmission in unpredictable and complex ways, especially when illuminated at high intensities or for longer durations (Mahn et al. 2016; Wiegert et al. 2017). Stimulation of axon terminals may even cause some back-propagated activation of indirect pathways influencing saccades (Inoue et al. 2015) or it can depolarise both inhibitory and excitatory neurons (Jazayeri et al. 2012). (Nassi et al. 2015) demonstrated this exact phenomenon, when attempting to optogenetically target excitatory neurons in the macaque V1, and indirectly exciting inhibitory neurons. As a means to overcome such a limitation, (Shewcraft et al. 2020) recently demonstrated the ability to optogenetically manipulate either excitatory or inhibitory activity alone, by carefully selecting stimulation parameters such as light pulse duration.

Marmosets are a promising animal model for studying how neural circuits give rise to certain complex brain states and behaviours, and this remains true for their use in optogenetic protocols. While optogenetic techniques have only recently been introduced in marmosets, the smaller size of marmoset brains (relative to other primates) is already showing to be a key advantage. Forelimb movements have been induced through blue-light optogenetic stimulation of the marmoset motor cortex (Ebina et al. 2019). Using a gene expression system that amplifies neuronal expression of a ChR2 opsin gene variant with fast kinetics, Ebina and colleagues stimulated the motor cortex through a relatively large cranial window. Given that this has previously been difficult to accomplish in macaques, due to the size of their motor cortex restricting light permeability through neural tissue, this demonstrates a unique benefit of the marmoset model.

The small brain size of marmosets is also showing promise for enabling long-range transduction of optogenetic constructs. (MacDougall et al. 2016) developed a novel method to induce rapid photo-stimulation in individual neurons, for several months, in awake behaving marmosets. They reported that along with successful transport of the virus, ChR2 opsins and fluorescent proteins were trafficked further along long-range pyramidal neuron projections in the marmoset, from the site of injection, enabling accurate tracing of neural circuits that are directly and causally activated during visual behaviours. In the months following injection, similar excitatory and inhibitory changes in neural activity during optogenetic stimulation in marmosets (MacDougall et al. 2016) as have been previously observed in mice (Sato et al. 2014) and macaques (Han et al. 2011).

**Fig. 4:**
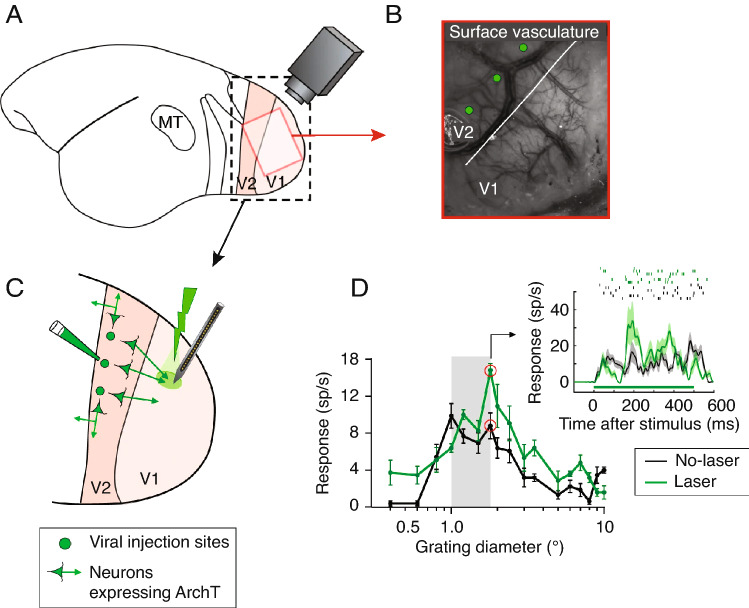
Optogenetic manipulation of V2 feedback projections to V1 in the marmoset. **A** Schematic of the optical imaging procedure on a marmoset brain, with V1 and V2 boundaries highlighted. Red box: approximate location of the optically imaged region. **B** Example optical image of the cortical surface vasculature imaged under green light, identifying the V1/V2 border (white line). Green dots: reference positions for viral injections carrying optogenetic constructs. **C** Schematic of the optogenetic inactivation paradigm, with recording array and laser photo-stimulation over V1. Green dots: viral injection sites in V2 matching optical image in B. Green arrows: ArchT-GFP expressing V2 neurons largely projecting back to V1. **D** Spatial summation curves for example V1 neuron recorded with (green) and without (black) laser stimulation. Grey area indicates the proximal surround of the V1 neuron. Inset: PSTH and raster plot measured at the stimulus diameters indicated by the red circles in the respective size-tuning curves of V1 neurons. Horizontal green line: laser-on time. Adapted from (Nurminen et al. 2018)

There have already been demonstrations of the success of optogenetics for investigating neural circuits in the visual cortex of marmosets. Recently, optogenetic suppression of the axon terminals of V2 feedback projections to V1, revealed an overall reduction in marmoset V1 responses to visual stimuli, as well as a reduction in surround suppression, a property fundamental to V1 (Fig 4**;** (Nurminen et al. 2018)). In macaques, optogenetic stimulation of the koniocellular compartment of the macaque LGN, while recording from V1, they observed short latency neural responses evoked in the supra-granular layers of V1, to which they are known to project anatomically (Klein et al. 2016). This study demonstrates that optogenetics is a suitable technique for studying longer-range projections. The relatively smaller brain size of the marmoset compared to the macaque suggests that it will be particularly suitable for studying network interactions across cortical distances, including between areas of the frontal and parietal cortices.

Finally, coupling optogenetics with a powerful imaging technique like two-photon microscopy, can further improve investigations into neuronal circuit mechanisms. Two-photon microscopy permits *in vivo* visualisation of neural activity at high resolutions, deep into the cortex and for extended periods of time. Briefly, it is a fluorescence imaging technique that relies on the principle of a fluorophore absorbing two photons of light and emitting a longer wavelength of light which is known to scatter less and penetrate deeper through neural tissue. Two-photon imaging is already well established in rodents (Holtmaat et al. 2009; Drew et al. 2010), mainly due to their relatively thin dura, upper cortical layers, and amenability to transgenic technology — features shared by marmosets. While it has been attempted in the areas most exposed to the cortical surface in macaques, like V1 (Stettler et al. 2006; Heider et al. 2010), penetration depths in terms of anatomical layers are more limited owing to the cortical thickness and vascularisation. To date, fewer studies have attempted two-photon imaging in marmosets, however, studies in somatosensory (Sadakane et al. 2015) and motor cortical areas (Ebina et al. 2018) have successfully imaged hundreds of neurons for several months, up to cortical depths of 500 μm (equating to layers 2/3) (Santisakultarm et al. 2016). Given the similarity in size and lissencephalic nature of the marmoset and mouse brains, it stands that the progress made in multi-area two-photon imaging in mice, using either multiple microscopes (Lecoq et al. 2014; Wagner et al. 2019) or a unique two-stage magnification process (Yang et al. 2019), is much more easily transferred to marmosets. That being said, to date, this technology has been limited to single area imaging in macaques and marmosets.

## Conclusion and future directions

Non-human primates have been essential in studying the neural mechanisms of cognitive behaviors, such as visual attention. However, there are still many open questions about how such behaviors arise from interactions across brain areas. New electrode technologies and molecular techniques like optogenetics continually increase the toolbox with which researchers can probe these questions. While macaques have historically been the dominant model in such investigations, given their extensive behavioural repertoire and our knowledge of their neurophysiology and neuroanatomy, the sulcal structure of their brain has been limiting in the application of such techniques. We propose that the marmoset monkey is the ideal primate model for this arsenal, and shows much promise to help bridge the gap between our anatomical knowledge of the structure of cortex and understanding how this precise architecture across brain areas gives rise to cognitive visual behavior. Combining optogenetic methods in marmosets with existing behavioural, multi-area neurophysiological, and neuroimaging approaches could help uncover the functional architecture underlying visual and cognitive behaviours in primates. However, because there is still a lot we do not know about marmoset neurophysiology and the extent of their behavioural capabilities, future research would greatly benefit from such investigations clarifying these gaps in our knowledge. Even if the current trajectory holds, and marmosets are restricted to more simple visual and cognitive behaviours, we argue that the benefits of their neuroanatomical and lissencephalic brain structure still provide important opportunities for the application of more complex techniques.
